# What if I die? Existential crises when caring for persons with severe mental disorders in rural South Africa

**DOI:** 10.1371/journal.pmen.0000288

**Published:** 2026-03-24

**Authors:** Olindah Silaule, Nokuthula Gloria Nkosi, Fasloen Adams

**Affiliations:** 1 Occupational Therapy Department, School of Therapeutic Sciences, Faculty of Health Sciences, University of the Witwatersrand, Johannesburg, South Africa; 2 Division of Occupational Therapy, Department of Health and Rehabilitation Sciences, Faculty of Health Sciences, University of Cape Town, Cape Town, South Africa; 3 Nursing Education Department, University of the Witwatersrand, Johannesburg, South Africa; 4 Division of Occupational Therapy, Department of Health and Rehabilitation Sciences, Faculty of Medicine and Health Sciences, Stellenbosch University, Stellenbosch, South Africa; PLOS: Public Library of Science, UNITED KINGDOM OF GREAT BRITAIN AND NORTHERN IRELAND

## Abstract

The role of informal caregiving is associated with various crises and psychological distress, including the caregiver’s fear of death and dying. A vast amount of evidence exists on the burden of care related to the psychological distress faced by the informal caregivers in mental health. However, there is a paucity of evidence on the existential crises faced by informal caregivers of persons with severe mental disorders. Exploring the existential concerns of informal caregivers is essential for understanding how fear of death contributes to their psychological burden, ultimately compromising mental and physical health. Such understanding is crucial for designing support strategies that foster resilience, reduce distress, and enhance caregivers’ overall health and quality of life. A descriptive qualitative approach was used. Semi-structured interviews were conducted with 12 informal caregivers who were purposely selected to participate in the study between January and June 2022. Informed, written consents for interviews and audio recording were obtained. Audio-recorded interviews were translated, transcribed, and analysed inductively on NVivo12 using thematic analysis. Two themes were identified, namely, navigating the challenges and distress of caregiving and caregivers’ experiences of existential death anxiety. Fear over the continuity of care in the event of their own death appeared to be closely linked to the death-related anxiety expressed by some informal caregivers. Such anxiety may compromise caregivers’ psychological well-being by heightening feelings of fear, helplessness, and uncertainty about the future, while also amplifying concerns regarding the capacity of people with severe mental disorders to function independently and receive consistent care within the community. Succession planning in caregiving is essential, with responsibilities shared among primary caregivers, families, and communities. Future studies should focus on developing and implementing stigma reduction programmes and caregiver support strategies strengthen coping, reduce burden, and promote the overall well-being of informal caregivers of persons with severe mental disorders.

## Introduction

Severe mental disorders(SMDs), including depressive disorders, schizophrenia and bipolar disorders, are chronic and counted among the top 20 disabling disorders [1,2]. They affect an estimated 4% of the adult population and contribute significantly to the global burden of disease, representing a major source of morbidity and disability worldwide [3,4]. In South Africa, SMDs account for approximately 26.1% of all reported mental health conditions [5]. While the exact prevalence of mental health disorders in Mpumalanga remains unclear, the province is reported to have one of the highest rates of mental health disorders in South Africa [6]. A profile of common causes of mental illnesses in Mpumalanga, a predominantly rural province, indicates that most patients are diagnosed with bipolar disorder, schizophrenia, major depressive disorder, and substance-induced psychosis [7]. Factors such as limited access to mental health services, socioeconomic challenges, and rural healthcare disparities may contribute to the high burden of serious mental disorders, making the province a relevant site for this study [6].

Globally, the growing burden of mental disorders has been overcome by persistent challenges, and one prominent challenge is the treatment gap between the need and provision of mental health services [8]. In high-income countries, about a third of the population with mental disorders receive treatment, but in LMICs, less than 5% of people with mental disorders receive treatment [9,10]. In South Africa, it is estimated that only 27% of individuals with SMDs receive treatment, with the dehumanising state of mental health services in rural areas often attributed to structural and systemic issues such as limited funding, poor policy implementation, and lack of infrastructure, limited institutional support, and staff shortages [11–13]. In a deinstitutionalised mental health system, the inadequate access to mental health services calls for informal caregivers to become key role players in the recovery of persons with mental disorders, particularly for those with SMDs who often require long-term care and management [14,15]. The role of being an informal caregiver is often occupied by family, friends, neighbours, or a good Samaritan, who are not remunerated for providing care, and who provide continuous care to a person with a SMD [16,17].

Informal caregivers are responsible for meeting the daily needs of the person with SMD, including financial support; providing emotional support; dealing with disruptive behaviour; monitoring their mental state; supervising medication; and identifying early warning signs of relapse and deterioration [15,18,19]. Dealing with the caregiving responsibilities has been linked to severe psychological distress and existential concerns among the informal caregivers [20]. Previous studies conducted in high-income countries and low and middle-income countries established that caring for persons with chronic conditions such as SMDs is a formidable task that has been linked with increased financial, emotional and physical burdens [16,21]. Various factors have been identified as contributing to these burdens. These include caregivers’ anticipation of negative health outcomes, grief over the lost potential of the care recipient, emotional distress from witnessing their loved ones’ suffering, as well as feelings of resentment, disappointment, and guilt arising from their perceived inability to alter the course of the illness [21–23]. The experience of burden, along with watching the care recipients go through the pain of dealing with their mental disorders, is likely to awaken existential death anxiety, which is brought about by the caregiver’s own fear of death and dying [21].

According to Yalom [24], humans possess a unique awareness of their own mortality, living with the understanding that death is an unavoidable part of existence. Death anxiety has been described as a multidimensional emotional response that emerges when individuals reflect on the reality of death, whether their own or that of others [25]. From an existential viewpoint, the awareness of life’s finitude profoundly influences how people construct meaning and confront uncertainty. For informal caregivers, ongoing exposure to a loved one’s mental or physical suffering may intensify this awareness, evoking distress related to both the potential loss of the care recipient and their own mortality [26]. This experience, conceptualised as existential death anxiety**,** represents a form of existential distress rooted in concerns about meaning, continuity, and the limits of human existence [26].

A study by Thakkar [27] reveals that parents of adult children with high and complex needs often face the question of what will happen to their children when they are dead. The concerns relate to whether their adult children would receive adequate care when they are no longer there to care for and advocate for them [27]. Dealing with this question is worrisome [27] as caregivers are confronted with the existential reality of their death and mortality. This has been shown to lead to increased levels of depression, hopelessness, powerlessness, and anxiety, which threaten the physical, emotional, and psychological well-being of caregivers [21,28,29].

Understanding the existential concerns is important for developing strategies for supporting the health and well-being of the informal caregivers of persons with SMDs. These concerns relate to deeper questions of meaning, purpose, and uncertainty about the future, which often underlie the emotional and psychological strain of caregiving. By recognising and addressing these issues, support strategies can help caregivers build resilience, find meaning in their roles, and cope more effectively with the ongoing demands of caring for a person with a severe mental disorder. Currently, limited studies report on the existential concerns, such as death anxiety, affecting informal caregivers in mental health. This study aims to address this gap by reporting on the existential crises experienced by informal caregivers of persons with SMDs in rural South Africa.

## Materials and methods

### Ethical statement

The University of the Witwatersrand Human Research Ethics Committee (M200957) and the Mpumalanga Department of Health Research Committee (MP_202010_010) provided ethical approval. The methods followed in this study were in accordance with the relevant ethical principles for medical research involving human subjects [30]. Before data collection, all participants were given an information sheet, which informed them about the aim of the study, confidentiality and anonymity. To ensure confidentiality, each participant was assigned a participant ID. Informed written consent was obtained from all participants to grant permission to participate and to record the audio of the interviews.

### Study design

A descriptive, qualitative design was used in this study. A qualitative approach allowed for an inductive analysis of reality as perceived by the individual participants, their life outlook, and their subjective experiences as informal caregivers of persons with SMDs [31]. Semi-structured interviews were conducted to record the experiences of the informal caregivers between January and June 2022. Data collected in this descriptive study were analysed systematically using a six-phase reflexive thematic inductive analysis approach proposed by Braun and Clarke [32]. The current article followed the Consolidated Criteria for Reporting Qualitative Research (COREQ) [33]

### Study context

The study took place in Bushbuckridge municipality in Mpumalanga province, South Africa. Bushbuckridge municipality lies in the north-eastern rural part of Mpumalanga and has a population of 750,821 [34]. The province is mostly rural, which makes it hard to attract skilled mental health professionals. Currently, there is no specialised psychiatric hospital in the province. There are two provincial hospitals and one district hospital that offer inpatient mental health services within the province. The two provincial hospitals have a bed capacity of 26 and 17, respectively. The district hospital has the largest mental health unit in the entire province, with a bed capacity of 50. As the largest mental health unit in the province, the unit caters for a large catchment area, including other parts of the province beyond the Bushbuckridge municipality, which was expanded by the introduction of forensic services since 2014. This mental health unit caters for both in- and outpatients with a variety of mental disorders, ranging from acute to chronic and forensic cases. People with SMDs, within Bushbuckridge municipality are often treated at this district hospital. Once discharged, People with SMDs, and their caregivers are referred to collect their psychotropic medication from their closest community health clinic, which varies in distance depending on where they live.

### Participants

The inclusion criteria for this part of the study were that participants (1) must be informal caregivers of a person with SMDs, including a family member, a friend, or any person for whom they provide unpaid care; (2) must be primary caregiver who have provided continuous care for longer than six months; (3) must be at least 18 years old; (4) must be performing caregiving duties at least once a week; and (5) participants had to present with either a subjective (emotional and psychological distress) or objective (observable and practical caregiving demands) burden, classified as mild, moderate, or severe, in the first study, which assessed levels of caregiver burden [35] and provide informed consent to participate in subsequent studies.

### Data collection

Potential participants were identified from the first study that aimed to establish the extent of burden among the informal caregivers of people with SMDs [35]. In total, 170 informal caregivers took part in the first study, of which 120 gave consent to be contacted for the subsequent PhD studies. Participants who presented with mild, moderate, or severe levels of subjective and objective burdens were purposely selected. During the first study, participants were informed of the second study, and their intention to participate was recorded.

The first author (OS), who served as the primary investigator (PI), contacted all potential participants telephonically, who confirmed willingness and availability to engage in the study. Before data collection, the PI attended a qualitative research methodology course to build capacity on semi-structured interviews and the use of NVivo 12 in qualitative data analysis. The PI (OS) conducted semi-structured interviews with the informal caregivers under the guidance of two research supervisors (FA and NGN). Interviews were conducted with 12 participants in their homes as preferred, which allowed flexibility to accommodate participants’ availability in their natural settings, where caregiving duties are mostly performed. The interviews were individual, with two interviews conducted with a parent couple and the mother and sibling of a care recipient. The duration of the interviews was between 30 min – 1hour, and on average, the interviews took 45 minutes to conduct. A demographic questionnaire was used to capture the characteristics of both the caregivers and the people with SMDs receiving care. During the semi-structured interviews, participants were invited to share their views on their experience of caregiver burden and the resources and support needed to address caregiver burden. The following open-ended question was used to start the conversation: Please can you tell me about your experience of caring for your family member (son/daughter/husband/wife/sibling/granddaughter/mother/father) with a mental disorder. This was followed by questions aimed specifically at exploring the distress among the informal caregivers; for example, ‘Describe the different burdens you have encountered since you started providing care to your family member/relative with a mental disorder’; ‘How have these burdens impacted your life?’; ‘What are the sources of burdens related to providing care to your family member/relative?’. Data collection continued until saturation was achieved. To determine data saturation, code frequency counts were used by reviewing each interview transcript after every interview and tracking the occurrence of new codes. Data saturation was considered reached when the frequency of new codes diminished [36]. Two written informed consents were obtained from the participants to data collection: one for participating in the study, and the other for audio recording. Participant identities were used to preserve the confidentiality of the participants. The interviews were conducted in Xitsonga, the participants’ home language. The interviews were audio-recorded, transcribed, and translated into English by a language specialist employed as a researcher at a research organisation based in the Bushbuckridge municipality. To prepare for data analysis, the PI listened to the audio recordings while simultaneously reviewing the transcripts. Any necessary corrections and omissions were made to ensure the accuracy of the data translation.

### Data analysis

NVivo 12 software was used in this study to analyse the data. To capture the experiences of the informal caregivers in their caregiving role, a six-phase reflexive thematic inductive analysis approach, proposed by Braun and Clarke, was used for systematic data analysis [32]. The following steps were followed: (1) Data familiarisation and writing familiarisation notes, (2) systematic data coding, (3) generating initial themes from coded and collated data, (4) developing and reviewing themes, (5) refining, defining, and naming themes, and (6) reporting [32]. For data immersion, the primary investigator (OS) repeatedly listened to the audio recording from each interview. This process was done concurrently with reading the transcripts to ensure the transcribed data reflected what the participants reported in the interviews. Familiarisation notes were made for each participant to demonstrate the patterns and meanings emerging from the data set. Codes were generated systematically by identifying segments of data that the PI assessed to be meaningful in relation to the phenomenon of interest, namely, caregivers’ experiences in each data item. The PI coded as many codes as many potential themes, and descriptions for each code were added to ensure consistency in the coding process. Notes were kept to help the PI reflect on some of the generated codes that needed further clarity and to help the researchers (OS, NGN & FA) think of potential themes. Duplicates were identified, and codes that needed clarity were identified as the grouping took place. This process enabled the researchers (OS, NGN & FA) to identify relationships in the generated initial codes. A codebook was generated to enable consistency and ensure that information was not missed. In addition, the codebook was used to identify relationships between the codes as guided by the allocated description. At this stage, some of the initial codes became themes. Additional codes, such as those that could not be grouped into a theme, were identified and grouped for further analysis in the next phase. Themes were refined by reading the coded data extracts for each theme, which ensured that these merged to form a coherent pattern that informed the specific theme. The PI then read the dataset and notes again to ensure the validity of the themes and to identify other codes that may have been missed. To ensure trustworthiness, an experienced qualitative researcher conducted an audit trail of the data analysis process. Trustworthiness was further ensured by presenting the analysis process and findings to the research supervisors (FA and NGN) and peers, which helped promote the quality and truthfulness of the findings. The PI conducted member checking by sharing the research findings with the participants. Due to the distance between the PI and the participants, this process was carried out via phone. The findings were summarised, and participants were invited to provide feedback on whether the findings reflected their experiences. They were also encouraged to share any additional thoughts or information they felt had not been fully captured. To ensure dependability, the PI kept a journal that allowed her to document thoughts and observations on the experiences of caregivers during the interviews and analysis process.

## Results

The informal caregiver sample was predominantly female (75%), with nearly half over 65 years (47.1%) and a third between 40 and 65 years (33.3%). Caregiving experience ranged from 5 to 20 years, with half providing care for 1–8 hours daily and 41.7% engaged for 19–24 hours per day (Table 1) [37]. Most caregivers (50%) were pensioners, while 33.3% were unemployed, with a household income of less than ZAR 5,000 ($274) per month. The people with severe mental disorders were mostly male (66.7%), primarily aged 30–39 years (41.7%), and unmarried (75%), with secondary-level education (75%). Most (58.5%) were unemployed and receiving a disability grant, clinically stable (83.3%), and over half were diagnosed with schizophrenia (58.3%) (Table 2) [37].

**Table 1:** Demographic characteristics of informal caregivers of persons with SMDs (n = 12).

Characteristic	Frequency	Percentage (%)
Age		
20–39	3	25.0
40–65	4	33.3
+ 65	5	41.7
Gender		
Female	9	75.0
Male	3	25.0
Marital status		
Single	2	16.7
Married	7	58.3
Divorced	2	16.7
Widower	1	8.3
Education		
Primary school level	2	16.7
Secondary school level	4	33.3
Tertiary level	2	16.7
Uneducated	4	33.3
Employment status		
Employed	1	8.3
Unemployed	4	33.3
Pensioner	6	50
Self-employed	1	8.3
Relationships		
Mother	6	50
Father	2	16.7
Sibling	4	33.3
Number of care recipients		
1	10	83.3
2	2	16.7
Duration of caregiving		
5–10 yrs.	5	41.7
11–20 yrs.	7	58.3
Daily caregiving (in hours)		
1–8 hrs.	6	50
9–18 hrs.	1	8.3
19–24 hrs.	5	41.7
Monthly household income (ZAR)		
≤ 1 000	1	8.3
1 100–5 000	10	83.3
≥ 5 000	1	8.3
Monthly medical costs (ZAR)		
≤ 100	1	8.3
101–500	11	91.7
Residence		
Same as care recipient	8	66.7
Different from the care recipient	4	33.3

**Table 2:** Demographic characteristics of the people with severe mental disorders (n = 12).

Characteristics	Frequency	Percentage (%)
Age		
18- 29	3	25
30 - 39	5	41.7
40 - 49	2	16.6
≥ 50	2	16.6
Sex		
Female	4	33.3
Male	8	66.7
Marital status		
Single	9	75
Married	0	0
Divorced	2	16.7
Widower	1	8.3
Education		
Primary school level	3	25
Secondary school level	9	75
Employment status		
Unemployed (without a social grant)	4	33.3
Unemployed (with social grant)	7	58.3
Pensioner	1	8.3
Diagnosis		
Schizophrenia	7	58.3
Psychotic disorder Not Otherwise Specified	2	16.7
Substance use disorder	2	16.7
Major depressive disorder	1	8.3
Disease status		
Relatively stable	10	83.3
Unstable	2	16.7
Number of hospitalisations in the previous year		
None	10	83.3
1–2 times	2	16.7
Number of family members living in the household		
1–5 people	8	66.7
6–10 people	4	33.3

The following themes were identified: Navigating the challenges and distress of caregiving, and caregivers’ experience of existential anxiety. Each theme comprises sub-themes providing a multifaceted and detailed breakdown of the findings.

### Navigating the challenges and distress of caregiving

When asked about their caregiving experiences in caring for people with severe mental disorders (SMDs), the participants described the significant responsibilities embedded in their roles. These responsibilities arose from an ongoing awareness of their duty to care, concerns about the well-being of the care recipients, and the challenges of managing their needs within the limits of their own capacity. Caregivers’ narratives highlighted the tension between the demands of caregiving and the practical and emotional challenges they face, including feelings of fear, obligation, and powerlessness in certain situations. In this theme, informal caregivers described their sense of responsibility and accountability in meeting the needs of people with SMDs. This sense of responsibility was driven by the need to ensure the health and well-being of care recipients, protect them from harm, and support their participation in daily life. These were expressed through the following sub-themes: (1) responsibility for health and well-being; (2) responsibility for meeting physical needs; and (3) responsibility for enabling participation in daily occupations. These sub-themes underscore the demands of caregiving and nature of responsibility that informal caregivers have when caring for a person with a SMD.

#### Responsibility for health and well-being.

Participants indicated having a sense of responsibility towards supporting the health and well-being of people with SMDs. Various responsibilities connected to supporting the health of the people with SMDs were identified, including supporting them in seeking help for their condition, visiting them while in a hospital, and managing their medication following discharge from the hospital. Because most people diagnosed with a SMD require assistance managing and maintaining their health, the informal caregivers felt they must fulfil this role to ensure they remain stable, as expressed by the following participant:

“Sometimes when he was walking around the yard, I would ask him what is wrong and he would say, ‘I cannot sit down; I have to keep walking because they are telling me to walk’. When I ask him whom are those people telling him to walk, he would not tell me. When his father came back from work, I would tell him everything that happened during the day, and he would suggest that we take him to the hospital. When we get to the hospital, they would give him an injection, and that is when he would sit down.” (Mother, age 70)

Participants expressed a sense of responsibility to ensure the well-being of not only the care recipient but also the other members of the family. To fulfil this responsibility, they must be accountable in their caregiving duties and play a protective role by becoming a shield for other members of the family. Protecting other family members becomes an obligatory role, as their sense of responsibility and fears force them to take up this role. The lack of choice led to some informal caregivers seeing themselves as the only person suitable to provide care and ensure the well-being of the people with SMDs. As a result, they self-sacrifice to ensure the other members of the family remain protected. This lack of perceived choice and the accompanying burden of obligation are central to the psychological distress, evoking guilt and a loss of personal freedom. A participant in the following comment explains this:

“I was sad. Our mother is alive, but I fear that he might find an object and beat her with it, and that is the reason why I took him with me. What can I say … he is my brother, and there is nothing I can do because I am the eldest; I have to care for him. I cannot give him to someone else to care for him and as I have said that, I fear that he might hurt our mother if he can go and stay with her. He was staying with my mother but we were afraid that he might hurt her because there are times where he is uncontrollable.” (Sister, age 37)

In an effort to safeguard the health and well-being of people with SMDs, informal caregivers also act as mediators between the care recipients and the community. Because of the nature of their mental conditions, some people with SMDs get into conflicts with members of the community, and as a result, the community members approach the informal caregiver to express their complaints regarding the conduct of their care recipients. The caregiver’s narrative illustrates how mediating conflicts and assuming financial responsibility for the actions of the person with an SMD may be emotionally draining and contribute to feelings of helplessness and isolation, as explained in the following comment:

“When he has had fights with people, they come to me and complain about what he has done to them. They cannot complain to him because they know that he is not okay. I have to deal with those situations. If I have to pay for whatever damage he has done, I pay. If I have to talk to those people in a polite manner, I do it as well and make them understand that he is mentally ill.” (Sister, age 37)

#### Responsibility for meeting physical needs.

The caregivers expressed a sense of responsibility to fulfil the physical needs of the people with SMDs. The tasks associated with fulfilling this responsibility include providing financial support, ensuring food security, managing the disability grant responsibly to meet the needs of the people with SMDs, and safeguarding them from potential exploitation by the community. Due to the disabling nature of their mental health conditions, most people with SMDs are unable to obtain and retain employment, and as a result, they tend to depend on their caregivers for financial support. These tasks were often carried out despite severe financial constraints. Such accounts reflect both the demands and responsibilities of caregiving, as meeting physical needs became intertwined with maintaining the dignity and survival of their loved ones. One participant said the following:

“We are all living on that little money I get from the government, and you can see that it is very challenging. As grown-up as he is, I still take care of him, and when I receive the pension money, I have to make sure that I buy him everything that he has to use at his house. I buy from maize meal, meat, and toiletries. When he does not have the washing powder, the bath soap, or the deodorant … I have to buy all of those things. Whatever it is that I eat here, he should have them as well at his house, and they are never enough. When we do not have food, I just go to the tuck shop around here and ask for food on credit so that I can give him. So … aaah!” (Mother, age 62)

In spite of the financial strain expressed by the informal caregivers, some had to make sacrifices to meet the financial demands of their care recipients to protect them from being exploited by the members of the community. Due to the limited autonomy, some people with SMDs are taken advantage of by community members who send them to do odd jobs, such as woodcutting or running errands, without proper payment. The informal caregivers feel helpless to preserve the dignity of their care recipients. They feel a sense of responsibility to protect people with SMDs from exploitation, which further adds to the financial strain faced by informal caregivers who must provide the money to prevent the care recipients from seeking work outside their home. This protective instinct highlights the tension between the caregivers’ desire to maintain control over the well-being of the people with SMDs and the feelings of powerlessness imposed by social and economic constraints, exemplifying the level of psychological distress experienced by caregivers and reflecting fear, a sense of responsibility, and ongoing emotional strain. The participants explained it as follows:

“Yes, they always looked for him. And we would always fight with those people and tell them that this person is not mentally okay for them to be sending him around. He is the person who always wants money, and he wants it almost every day. And because he is in that condition and we have to ensure that we meet him halfway with whatever he wants because if we do not do that, it will trigger something in his head. We are making sure that we give him what he wants because we don’t want him to think of doing petty jobs like chopping woods for people in return for money.” (Brother, age 42)

“People here in the village used to give him odd jobs like fetching wood in the bushes, and they would pay only R10 ($0.54) for that, or they give him a jar of traditional beer as the payment for fetching the wood. A mentally ill person does not know the worth of the work he has done; he just accepts anything.” (Mother, age 66)

Responsibility for enabling participation in daily occupations. The participants expressed feeling responsible for ensuring the people with SMDs’ participation in daily activities, which is linked to performing daily tasks for them, providing constant supervision, and structuring their daily routines. The caregivers expressed a sense of responsibility for assisting people with SMDs with daily activities, including cleaning, cooking, and washing. In addition, the caregivers said they perform activities to prevent people with SMDs from harming themselves. These actions stemmed not only from practical necessity but also from an underlying anxiety about losing control or failing in their caregiving role. One participant explained it as follows:

“My responsibility as a caregiver is to make sure that he eats proper food, take the medications. I wash his laundry so that he can be neat around other people, and he does not look different to other people. I do that to make sure that he is well cared for and he takes his medications on time. He is able to do many things on his own, but I cannot trust him with cooking because I fear that he might attempt to cook on the stove he gets burned, so I cook for him. I also clean his room because he leaves it untidy most of the time.” (Sister, age 37)

The participants also explained that they must structure the people with SMDs’ daily activities and provide constant supervision. Without such structure, these individuals often fail to participate in their daily occupations as expected, requiring caregivers to provide guidance and organisation. In the absence of such structure, caregivers felt anxious and responsible, often at the cost of their own autonomy. A participant explained it as follows:

“I am taking care of him, and I have to make sure that I remind him to do things such as bathing; remind him that he did not bath yet and he has to do it. So, it is difficult because I always have to look after him like a little child. He bathes by himself, and he also does his own laundry when I tell him to. If I do not tell him, he does not do it. Yes, I always have to give instruction so that he can do what he should do. If maybe, I have to go somewhere and I leave without telling him what to do, he does not do anything.” (Mother, age 42)

### Caregivers’ experience of existential death anxiety

The informal caregivers said they experience existential anxiety in their caregiving role. This theme describes their fear and anxiety over the future of their care recipients when they die, and is divided into two sub-themes, namely (1) experiencing fear about what will happen to a person with a SMD in the event of their caregiver’s death; and (2) experiencing fear for the future of people with severe mental disorders due to the unpredictability of their condition and their ability to earn a living. These sub-themes highlight the intense emotions that the informal caregivers deal with because of the uncertain future of the functioning and continuity of care for people with SMDs.

#### Experiencing fear about what will happen to a person with a severe mental disorder in the event of their caregiver’s death.

Participants were anxious about what would happen to the people with SMDs they are caring for in the event of their death. These fears are connected to existential death anxiety, continuity of care, and concerns over whether people with SMDs would be able to access the help they need if their caregiver died. Existential death anxiety may arise from concerns that care for the people with SMDs could be disrupted if the caregiver were to die. Because of the level of dependency and many responsibilities attached to the caregiver role, the caregivers feel distressed and helpless because they fear other members of the family will struggle to cope with the demands of caregiving. One participant expressed his fears as follows:

“We all know that we are not here to live forever, and my worry is that if we die there will be no one to look or care for him. I do not think his brothers and sisters would be able to care for him because they would be married and they will not be able to take care of an older person like him. That thought does not give me peace of mind because I know that one day we will die. That is the most challenging for me, and I do not know what to do about it.” (Father, age 72)

The caregivers feared that the people with severe mental disorders would not receive the same level of care they provide as primary caregivers, and that, in their absence, these care recipients would struggle to access the medical care necessary to remain stable. This preoccupation with their own mortality and the continuity of care is largely attributed to the lack of independence of the people with SMDs, their constant need for supervision, and the perception that other family members are less equipped to meet the demands of caregiving, as illustrated in the following comment:

“Another thing is that when I am caring for him, I always wonder how he will live if I die because he does not know when he has to go to the hospital. Sometimes I wonder if my closest family will be able to take over and care for him when I am no longer around. I always ask myself these questions. There is definitely no one who will be able to care for him the way I do. I am just wondering if that particular person who takes over will be able to be patient enough to make those trips to the hospital or not. I am just asking myself that question.” (Mother, age 42)

While some participants expressed being content with their ability to fulfil their responsibilities to provide for the daily needs of the people with SMDs, they were anxious over whether these needs would be met in the event of their death. One participant explained as follows that they are constantly worried and under pressure to ensure they provide the best care possible to make up for their absence in the future:

“My biggest worry is that he will have no one to care for him when I am no more. Now that I am still alive, I am able to care for him, and there is no day that he goes to bed hungry. When he goes out, I make sure that I know where he is and if he has eaten something. It affects me a lot because I do not know how long I will live, and I do not know who will help him when I am no more.” (Mother, age 70)

The informal caregivers said that managing the disability grant of the people with SMDs gave them a sense of responsibility for the care recipients. Since most caregivers were solely responsible for both the individual and the management of their funds, participants expressed apprehension and anxiety over the potential mismanagement of the grant in their absence, and the possible consequences for the quality of care the person with SMD would receive.. This demonstrates the sense of ownership that caregivers feel towards their caregiving duties, which can be seen as an act of selflessness or sense of mistrust towards the other family members in fulfilling this role. A participant explained her apprehension as follows:

“Whatever it is that they do, I have to see it including the mistakes that they make because my sister cannot always come home just to see how they are doing. I am the one responsible for them because I am the one who is receiving their disability grant. My worry is that if I die, they will have no one to manage their money well because people are not the same even if they are siblings. You may find that the person who would take over would not be as responsible as I am with their money. Someone can take over from me as their caregiver but they may not be able to care for them the way that I do.” (Sister, age 37)

Experiencing fear for the future of people with severe mental disorders. The participants highlighted their fears around the unpredictability of the conditions of people with SMDs, anticipated future relapses, and the ability to earn a living in the future. The informal caregivers reported living in a state of constant fear regarding future relapses, which contributed to ongoing anxiety and emotional strain. The caregivers expressed considerable doubt and mistrust regarding the mental state of people with severe mental disorders, even when they were relatively stable on medication. Additionally, caregivers reported feelings of hopelessness, as they did not believe the destructive behaviours of the care recipients would change, a perception attributed to the chronic nature of SMDs, which predisposes them to multiple relapses. One participant described their fear as follows:

“He was a normal person and he was working. He was able to buy whatever he wanted, but he has destroyed everything that he bought while he was working. Now, I do not even have the courage to buy him a cheaper bed because when I think about it, I think that maybe he will destroy it. So, I am very discouraged to buy him things that he needs because I am scared that he can destroy [it] in a blink of an eye.” (Mother, age 62)

The caregivers were also concerned about the ability of the people with SMDs to earn a living in the future Because the informal caregivers are often the sole providers of care and the people with SMDs are highly dependent on them, caregivers worried that their care recipients would suffer in the event of their death. One participant described their hope that their care recipient would find a job to secure an income as follows:

“I have accepted everything, but my worry is that he will suffer if I die. It would make me the happiest person alive if I were to hear that he has found a job. Even if it is not a fancy job, it would be fine. Any job where he can come home on weekends. It will help me because even if I die, I will know that he has something to do for a living.” (Mother, age 71)

## Discussion

This study aimed to explore the lived experiences of informal caregivers of persons with SMDs. The findings revealed two main themes, namely navigating the challenges and distress of caregiving and their experience of existential death anxiety while occupying the caregiving role. The theme of navigating the challenges and distress of caregiving had three sub-themes that highlighted the sense of responsibility and accountability the informal caregivers have towards ensuring the health and well-being of the people with SMDs, meeting their physical needs, and enabling their participation in daily occupations. The two sub-themes of caregivers’ experience of existential death anxiety were fear for the future of people with SMDs if the caregiver died, and concerns about the unpredictability of their mental conditions and their ability to earn a living in the future. In these themes and sub-themes, the experiences of informal caregivers were characterised by fear, persistent worry, despair, and feelings of powerlessness, reflecting the tension between the demands of caregiving, their sense of responsibility and ownership over the care of people with SMDs, and their need to protect and ensure the well-being of their care recipients.

Most informal caregivers (41.7%) in this study are in their late adulthood, while many others are in middle adulthood (33.3%). The majority are parents of people with SMDs (66.7%), while a notable proportion are siblings (33.3%). These relationships, together with caregivers’ stage of life, may predispose them to experiencing psychological distress related to their sense of responsibility. The informal caregivers perceived their sense of responsibility and accountability towards meeting the needs of the people with SMDs, which appeared to be driven by their fear, the need to be protective of the well-being of their care recipients, and their powerlessness brought on by their existential death anxiety. Erikson’s [38] human development theory classifies middle adulthood as a stage of generativity versus stagnation that is characterised by the need to give to and assume responsibility for others. For parents, generativity is achieved by successfully nurturing their children’s growth and development to help them become capable and contributing members of society. Successfully achieving generativity is therefore linked with life satisfaction and psychological well-being [39]. Parents of people with SMDs are the primary source of care and support for their children, despite age, and as a result, active parenting does not end even when the care recipients reach adulthood. Subsequently, the prolonged care required by people with SMDs is often accompanied by strain that threatens the caregivers’ psychological well-being [40]. An important finding from this study is that a notable proportion of caregivers were siblings (33.3%). While parents often assume caregiving responsibilities due to cultural expectations and family roles, siblings may take on this responsibility either as co-caregivers or as primary caregivers when parents are aging, unavailable, or deceased. Unlike parental caregivers, siblings are frequently in the midst of their productive life stages, balancing employment, child-rearing, and other familial responsibilities alongside their caregiving responsibilities [41,42]. This could result in a dual burden, which could increase the risk of emotional distress, financial strain, and role conflict. Vukeya et al. [41] reported that siblings of people with SMDs in rural South Africa experienced caregiving as overwhelming and emotionally destabilising, with an intense need for structured support. Similarly, Young and Flannigan [42], highlighted that siblings faced significant financial strain, stigma, and uncertainty about their future when caring for a brother or sister with mental illness. These findings align with the experiences of the caregivers in our study, highlighting the unique challenges they face in balancing caregiving with their own developmental and social responsibilities. This, therefore underscores the importance of providing targeted support and interventions, particularly in low-resource settings such as rural South Africa.

In this study, most care recipients (66.7%) are in early adulthood, and some (33.3%) are in middle adulthood. While many were considered to be relatively stable, i.e., not showing any positive psychiatric symptoms, they still displayed dependency towards their informal caregivers. As a result, the informal caregivers feel a sense of responsibility to ensure the health and well-being of the people with SMDs and to meet their needs. Consequently, fulfilling these responsibilities results in psychological distress due to the sense of responsibility arising from the caregivers’ knowledge of the functional limitations of the people with SMDs leads to fear, worry, and feelings of powerlessness when they face their own mortality. According to Erikson, generativity is characterised by the psychological “need to be needed”, and therefore, the role of caregiving can be related to higher perceptions of generativity [43]. The caregivers in this study expressed a sense of responsibility towards the people with SMDs, and they often identified themselves as the sole person suitable for fulfilling the role of caregiving. While assuming ownership of their caregiving duties can result in satisfaction for caregivers and promote the well-being of the people with SMDs [44], it can also be detrimental to caregivers’ psychological well-being. For some caregivers in this study, fulfilling the role of caregiving is obligatory and means dealing with financial strain and protecting both the care recipient and other members of the family, which eventually leads to self-sacrifice. This self-sacrifice leads informal caregivers to sacrifice the little money they have to meet the financial needs of the people with SMDs to protect them from exploitation by the community. According to Van Nistelrooy [44], the act of self-sacrifice tends to be inherent in the caregiving role because caregivers are faced with a constant need to sacrifice their time, finances, relationships, and at times, their employment to help improve and protect the well-being of their care recipients [45]. The limited autonomy in caregiving can leave caregivers feeling constrained by external circumstances, reducing their sense of agency in managing both the care recipient’s needs and their own well-being [46]. Consequently, occupying the caregiving role predisposes participants to a state of powerlessness, which likely leads to feelings of stagnation. These findings are similar to those of Jönsson [47], who found that caregivers of people with mental disorders experience worry and powerlessness while fulfilling their caregiving responsibilities.

The experience of existential death anxiety expressed by the informal caregivers in this study is expected because most (41.7%) are in late adulthood. Late adulthood is the last stage of life, and individuals in this stage are confronted with the reality of death and their mortality. Erikson identifies late adulthood as a stage of ego integrity versus despair [48]. Despair surfaces when older individuals are confronted with stressful life situations [49]. The demands of caregiving, such as being the sole provider of care for their adult children, dealing with financial strain, dealing with the functional limitations of their care recipients, and the unpredictability of SMDs, trigger a state of despair among caregivers. Consequently, caregivers experience an existential crisis brought on by their concerns that the person with SMD will suffer when the caregivers’ care ceases in the event of their death. Most care recipients (58.3%) in this study developed schizophrenia in early adulthood and are unmarried. Although they are in their productive stages of life, these people with severe mental disorders remain dependent on the care and structure provided by their caregivers to function in their environment. This is because schizophrenia is a chronic mental disorder characterised by psychosis and unpredictable, hostile, or risky behaviour with long-term functional limitations [50,51]. It is therefore understandable that the informal caregivers in this study expressed fear regarding the unpredictability of the conditions of people with SMDs, anticipated future relapses despite stability on treatment, and concerns about their ability to earn a living in the future. Given the chronic nature of schizophrenia, those diagnosed tend to be susceptible to relapses even while on treatment [35]. In fact, the relapse rate of schizophrenia is estimated to range between 50% and 92% globally [52]. It is therefore a serious concern that care for these people with SMDs relies on the elderly, who are themselves a vulnerable population. In addition to coping with a decline in their own health, they must bear the stressful demands of caregiving, which poses a threat to their psychological well-being [37]. Findings from this study suggest that stress among the elderly population may act as a predisposing or precipitating factor for despair [49], which can be attributed to the existential crisis experienced by the caregivers.

Most of the informal caregivers in this study are the sole providers of care for the people with SMDs, and their concerns about the continuity of care in the event of their death highlight issues related to succession planning for caregiving in rural South Africa. A study by Thakkar [27] on succession planning for adult children with disabilities revealed that caregivers often avoid future planning for their care recipients because they find this topic overwhelming. This is largely because they are forced to deal with the inevitability of their own mortality and deal with the anxieties of relinquishing their responsibilities to others, which may mean an end to their caregiving role [27,53]. The caregivers in this study identified themselves as ‘the only person fit to provide quality care’, highlighting a lack of confidence in the other family members’ ability to provide quality care for the people with SMDs. This perception appeared to stem from a deep sense of responsibility, emotional investment, and a sense of ownership over the care of the care recipient, as well as mistrust regarding financial issues and decision-making within the family. Caregivers expressed concern that other family members might not prioritise the care recipient’s well-being to the same extent or might lack the patience, understanding, and commitment required to manage the complex demands of caregiving. These reflections reveal how feelings of mistrust and ownership shape caregivers’ reluctance to delegate care and underscore the relevance of succession planning in such contexts. Nonetheless, the potential consequences of the absence of a succession plan remain significant and cannot be overlooked. In the event that the primary caregiver dies or ceases to provide care, the absence of a succession plan can lead to crises and emotional trauma for everyone involved, and cause unexpected dilemmas for other members of the family who may be ill-equipped to occupy this role [54]. In rural South Africa, where there is a limited institutional care for people with SMDs [53,54], the death of a primary caregiver may pose a serious risk for recurrent relapses and even homelessness. A study by Moyo et al. [55] conducted in South Africa reports that the death of a parent is a risk factor for homelessness among mentally ill persons.

Succession planning with clear action steps is necessary to ensure continuity of care for people with SMDs in the future. This may involve structured family meetings facilitated by mental health professionals, such as social workers or community-based mental health professionals, to support discussions about future care arrangements, clarify caregiving roles, and document responsibilities that the family has agreed to. Incorporating succession planning into discharge and follow-up processes could help families prepare for the delegation and transition of caregiving responsibilities. Training for community-based mental health professionals can help them facilitate these conversations, given the emotional and relational challenges that often accompany caregiving.

The continued stigma surrounding mental illness in communities remains a serious problem with substantial consequences for people with SMDs and their caregivers [56]. As highlighted in the findings of this study, conflicts with and exploitation by members of the community were reported to contribute to the strain of caregiving. This indicates that caregivers’ preoccupation with their care recipients’ well-being extends beyond the immediate demands of caregiving to concerns about the potential worsening of social conflicts, exploitation, and stigma in the community in the event of their death. The findings of this study are similar to those of previous studies conducted in rural South Africa and rural Canada that report that exploiting people with SMDs is common in rural and remote areas [56,57]. The families of people with SMDs are often blamed for the behaviour of their family members with mental illness and endure exploitation from community members who demand payment for damages caused by the care recipients [56,57]. These findings suggest that the ill-treatment that the caregivers witness their care recipients experience from the community heightens their preoccupation with concerns over the well-being of people with SMDs in the event of their death. This broader context amplifies the existential death anxiety experienced by caregivers, as they anticipate the challenges their care recipients may face and the potential disruption in care, reinforcing the intensity of their sense of responsibility and the weight of their caregiving role.

### Strengths and limitations

This study highlighted the need to investigate the impact of providing care to persons with SMDs on the health and well-being of elderly caregivers, particularly in rural areas where caregiving is largely shouldered by this vulnerable group. It underscores the importance of developing strategies to distribute caregiving responsibilities among primary caregivers, their families, and the broader community. As a qualitative study with a small, homogeneous sample, the findings are not generalizable to the broader population of informal caregivers of people with SMDs in the Bushbuckridge municipality. This study formed part of a larger PhD project and followed a quantitative study that evaluated the extent of burden within the same population. Consequently, only participants who reported caregiver burden were selected, which introduces a selection bias by excluding caregivers without perceived burden from the study.

## Conclusion

The findings of this study highlight the presence of existential crises among the informal caregivers, as evidenced in the distress and existential death anxiety established in this study. The challenges experienced by informal caregivers of persons with SMDs are multidimensional; therefore, interventions aimed at alleviating caregiver burden require multisectoral collaboration between the health sector, social development, local government, and community organisations. This requires immediate attention, as strategies for the care and support of informal caregivers should be developed and implemented to enable them to cope with the demands of caregiving.

Such strategies may include psychoeducation and support group interventions to enhance caregivers’ knowledge, provide peer support, and reduce feelings of isolation. Mindfulness-based approaches can be incorporated to help caregivers manage stress and emotional distress, while empowerment-focused programmes can strengthen caregivers’ confidence and agency in navigating their roles. Collectively, these strategies should equip informal caregivers with the knowledge and skills to support people with SMDs in structuring and performing daily activities, thereby facilitating the care recipients’ independence and reducing caregiver burden. Furthermore, caregivers should be empowered to take an active role in the planning and delivery of mental health services to ensure their appropriateness and sustainability, while also promoting caregivers’ own health and well-being.

On the policy level, the findings call for integration of caregiver support into national health frameworks, the needs of the caregivers including existential concerns, should be formally recognised in national mental health policies. Providing fee-free mental health services and integrating caregiver support into healthcare plans can reduce economic and psychological burdens that contribute to distress [58,59]. Policymakers should include informal caregivers as key stakeholders, particularly in low-resourced settings like rural South Africa, to ensure resources and services address the needs of both care recipients and caregivers. Adequate budgeting is essential to support effective implementation. There is a need for structured family-based interventions and succession planning, where family interventions, which can be facilitated by community-based mental health professionals, can clarify caregiving roles, document future care plans, and reduce anxiety about care continuity. Training of these mental health professionals should be provided to guide these discussions sensitively, considering the emotional and relational complexities involved. Participatory and task-shifting approaches, such as involving caregivers in the development of intervention programmes and policy can enhance agency and reduce feelings of helplessness. Task-shifting, where trained lay or mid-level workers such as community health workers deliver caregiver-oriented services, can help improve access and support within communities while also addressing the existential death anxiety experienced by caregivers. By providing guidance, emotional support, and tangible assistance, these workers can help caregivers manage fears about the continuity of care, reduce feelings of helplessness, and promote a sense of security regarding the well-being of their care recipients. Respite services and caregiver support programmes, including psychoeducation, support groups, mindfulness, and empowerment, can help reduce stress and prevent emotional exhaustion. In LMICs, formalising these services through policy development and implementation is essential to ensure sustainable, accessible, and equitable support. Embedding caregiver-focused interventions into national mental health strategies and allocating resources for their delivery can help address the multidimensional burdens caregivers face, including economic, emotional, and existential challenges such as death anxiety. Reducing stigma and improving access to community-based mental health services can reduce caregivers’ emotional and practical burdens, including existential death anxiety linked to concerns about care continuity. Policies should prioritise anti-stigma campaigns and integrate caregiver-focused mental health support into national and local health frameworks. Adequate financial and human resource allocation is essential to ensure caregivers receive appropriate support and that the needs of care recipients are met.

Given the exploratory nature of this qualitative study, future quantitative research is needed to measure the prevalence, intensity, and predictors of existential death anxiety among informal caregivers of people with SMDs. Such studies would enable generalisation of findings to broader rural South African populations. Studies should investigate succession planning and the role this plays in ensuring continuity of care following the death of the primary caregiver in mental health. Focus should also be directed towards investigating stigma reduction programmes to help reduce stigma towards people with SMDs and their caregivers.
